# Elevated Bone Turnover Markers after Risk-Reducing Salpingo-Oophorectomy in Women at Increased Risk for Breast and Ovarian Cancer

**DOI:** 10.1371/journal.pone.0169673

**Published:** 2017-01-06

**Authors:** Ingrid E. Fakkert, Eveline van der Veer, Elske Marije Abma, Joop D. Lefrandt, Bruce H. R. Wolffenbuttel, Jan C. Oosterwijk, Riemer H. J. A. Slart, Iris G. Westrik, Geertruida H. de Bock, Marian J. E. Mourits

**Affiliations:** 1 Department of Epidemiology, University of Groningen, University Medical Center Groningen, Groningen, The Netherlands; 2 Department of Laboratory Medicine, University of Groningen, University Medical Center Groningen, Groningen, The Netherlands; 3 Division of Geriatric Medicine, Department of Internal Medicine, University of Groningen, University Medical Center Groningen, Groningen, The Netherlands; 4 Division of Vascular Medicine, Department of Internal Medicine, University of Groningen, University Medical Center Groningen, Groningen, The Netherlands; 5 Department of Endocrinology, University of Groningen, University Medical Center Groningen, Groningen, The Netherlands; 6 Department of Genetics, University of Groningen, University Medical Center Groningen, Groningen, The Netherlands; 7 Department of Nuclear Medicine and Molecular Imaging, University of Groningen, University Medical Center Groningen, Groningen, The Netherlands; 8 Department of Biomedical Photonic Imaging, University of Twente, Enschede, The Netherlands; 9 Department of Gynecology, University of Groningen, University Medical Center Groningen, Groningen, The Netherlands; Medical University of South Carolina, UNITED STATES

## Abstract

**Background:**

Risk-reducing salpingo-oophorectomy (RRSO) reduces ovarian cancer risk in *BRCA1/2* mutation carriers. Premenopausal RRSO is hypothesized to increase fracture risk more than natural menopause. Elevated bone turnover markers (BTMs) might predict fracture risk. We investigated BTM levels after RRSO and aimed to identify clinical characteristics associated with elevated BTMs.

**Methods:**

Osteocalcin (OC), procollagen type I N-terminal peptide (PINP) and serum C-telopeptide of type I collagen (sCTx) were measured in 210 women ≥ 2 years after RRSO before age 53. BTM Z-scores were calculated using an existing reference cohort of age-matched women. Clinical characteristics were assessed by questionnaire.

**Results:**

BTMs after RRSO were higher than age-matched reference values: median Z-scores OC 0.11, p = 0.003; PINP 0.84, p < 0.001; sCTx 0.53, p < 0.001 (compared to Z = 0). After excluding women with recent fractures or BTM interfering medication, Z-scores increased to 0.34, 1.14 and 0.88, respectively. Z-scores for OC and PINP were inversely correlated to age at RRSO. No correlation was found with fracture incidence or history of breast cancer.

**Conclusions:**

Five years after RRSO, BTMs were higher than age-matched reference values. Since elevated BTMs might predict higher fracture risk, prospective studies are required to evaluate the clinical implications of this finding.

## Introduction

Women from families with a high incidence of breast and ovarian cancer (hereditary breast and ovarian cancer; HBOC) have increased risks of both these cancers, especially women with a germline mutation in the *BRCA1* or *BRCA2* genes [1,2]. Risk-reducing salpingo-oophorectomy (RRSO) is advised to all *BRCA1* and *BRCA2* mutation carriers between age 35–40 and 40–45 respectively [3,4]. It is hypothesized that surgical menopause as induced by premenopausal RRSO increases fracture risk more than natural menopause, because of earlier age at menopause and acute and complete cessation of ovarian hormone production [5,6].

Current practice to identify women at risk of developing fractures is measurement of bone mineral density (BMD) by Dual-Energy X-ray absorptiometry (DXA) [7]. Previous studies on BMD and fracture incidence after surgical menopause provided conflicting conclusions, as some suggested lower BMD and higher fracture incidence [5,8,9], while others did not find a difference compared to age-matched controls [10,11]. Assessment of bone turnover by measuring bone turnover marker (BTM) levels after RRSO may be a useful addition to BMD measurement. BTMs may provide information on the influence of RRSO on both bone formation and resorption [12]. Furthermore, BTMs in blood or urine might predict fracture risk independently of BMD [12,13].

It has been shown that BTMs increase rapidly within one month after surgical menopause and remain increased until at least one year after surgery [14–17]. Bone resorption marker levels seem to increase faster than bone formation markers [14–16,18], but after several months to years, their ratios appear to normalize [14–18]. Studies comparing BTMs after surgical and natural menopause report conflicting results; one study showed increased resorption marker levels after surgical menopause compared to natural menopause, while others found no differences in BTMs between the groups [19–21]. Therefore, we compared BTMs in a group of women ≥ 2 years after RRSO at premenopausal age to age-matched reference values. Furthermore, we aimed to identify factors that characterize women with elevated BTMs after premenopausal RRSO.

## Methods

### Study population and protocol

At the University Medical Center Groningen family cancer clinic, all women with HBOC or *BRCA1/2* mutations have been registered since 1994 [4]. Between February 2011 and May 2012, all women with HBOC or *BRCA1/2* mutations who had undergone RRSO before the age of 53 at least two years before, were invited for osteoporosis screening. Women filled in a questionnaire and were screened according to a protocol including measurement of height, weight, collection of blood samples, and BMD measurement. Results on standard diagnostic procedures for osteoporosis in this study were described elsewhere [11]. Two women were excluded from the current analyses because their BTMs were not measured. All women gave written informed consent for inclusion in the study.

The institutional ethics review board of the UMCG stated that the study did not fall under the scope of the Medical Research Involving Human Subjects Act as the study was considered as a part of standard care. A waiver for ethical approval was provided. IGW, IEF, MJEM, EMA, JL and EV provided individual patient care and were involved in data collection for this study. These authors had access to identifying patient data during data collection as they were involved in patient care. The other authors had no access to the identifying patient data.

Reference data for BTM levels were retrieved from an existing local reference cohort of 350 healthy Dutch women. Menopausal status of reference women aged ≤ 50 years was not known. Reference women aged > 50 were ≥ 5 years postmenopausal with serum 25(OH)D3 (25OHD) levels >50 nmol/L and LS and hip BMD T-score > -2.5.

### Laboratory assessments

Non-fasting blood samples were obtained between 9:00 a.m. and 4:30 p.m. Serum samples were stored within 1 hour after collection at -20°C until analysis. Calcium and albumin were measured by colorimetric assay (Roche Modular P, Mannheim, Germany; inter-assay coefficient of variation (IE–CV) < 2.0% and < 1.8%; lower detection limit 0.05 mmol/L and 10 g/L for calcium and albumin respectively). Phosphate was measured by photometric UV assay (Roche Modular P, Mannheim Germany; IE-CV< 2.6%; lower detection limit 0.1 mmol/L). Serum 25OHD was measured by isotope dilution-online solid phase extraction liquid chromatography-tandem mass spectrometry [22]. Method specifications were: level of quantification 4.0 nmol/L; IE-CV < 14.1%; recovery 93–98%; linearity r^2^ = 0.997. Accuracy was secured by the use of reference material from the National Institute of Standards & Technology (Gaithersburg, MD). Serum 25OHD was considered low when < 50 nmol/L between October and April and < 75 nmol/L between April and October. Thyroid stimulating hormone (TSH) was measured by electrochemiluminescence immunoassay (Roche Modular E; Mannheim, Germany; IE-CV < 2.3%; lower detection limit 0.005 mU/L). PTH was measured by immunoluminometric assay (Cobas e 601, Roche; Mannheim, Germany; IE-CV < 3.2%, lower detection limit 0.040 pg/L).

Bone turnover was assessed by measurement of the levels of bone formation markers osteocalcin (OC), intact procollagen type I N-terminal propeptide (PINP) and bone resorption marker serum C-telopeptide of type I collagen (sCTx).

OC was measured by immunoradiometric assay (BioSource Europe S.A, Nivelles, Belgium; IE-CV 9.4%). OC levels were expressed in μg/L and Z-scores to correct for the influence of age. PINP levels were measured by using a radioimmunoassay (Orion Diagnostica, Espoo, Finland; IE-CV 9.0%) PINP levels were expressed in in μg/L and Z-scores. sCTX was measured by Electro-chemiluminescence immunoassay (Elecsys 2010; Roche, Mannheim, Germany; IE-CV 10.8%). sCTx levels were expressed in pg/ml and Z-scores.

### Clinical measurements

Fractures and risk factors for osteoporosis were assessed by questionnaire, based on the questionnaire used at our fracture and osteoporosis outpatient clinic [23]. The questionnaire was sent to the women before the visit and missing or inconsistent answers were discussed and corrected if indicated during the visit.

BMD of the lumbar spine (LS; anterior-posterior projection at L1–L4) and femoral neck (FN) were measured by DXA using a Hologic Discovery A densitometer (Hologic Inc., Bedford, MA). BMD was expressed in grams per cm^2^, BMD Z-scores and T-scores. BMD Z-scores present the number of standard deviations (SD) that an individual’s BMD differs from the mean BMD in a cohort of white age-matched women. T-scores present the numbers of SD from the mean peak BMD in women aged 20–30 years. Z- and T-scores were retrieved from the Hologic DXA machine and calculated using the standard reference databases for Hologic in the Netherlands were used [24,25]. A T-score ≤ -2.5 was considered as osteoporosis, based on the lowest T-score of either LS or FN.

### Statistical analysis

Results were expressed as median (inter quartile range [IQR: 25^th^ and 75^th^ percentile]) for continuous data and as number (%) for dichotomous data. BTM Z-scores were used to correct for the influence of age on bone turnover marker levels. A BTM Z-score represents the number of SDs an individual's BTM level differs from the mean BTM level in a cohort of age-matched women. Z-scores were calculated by the following formula: (BTM value of individual patient–mean BTM value /SD of matched 10-year reference cohort. The difference between PINP and sCTx Z-scores was calculated to determine the relative difference in bone formation and resorption. To compare BTMs after RRSO with BTMs in age matched controls, median Z-scores for BTMs were compared with a hypothetical median Z-score of 0 in the general population using Wilcoxon’s signed-rank test. Medians were used as BTM Z-scores did not follow a normal distribution in our study cohort.

To identify factors associated with elevated BTM Z-scores, linear univariate and multivariate regression analyses were performed. Women with recent fractures (i.e. within 12 months before BTM level measurement) or BTM affecting medication (current use of HRT or aromatase inhibitors (AI) or ever use of anti-osteoporotic drugs (AOD)) were excluded from the regression analyses, because these factors are supposed to strongly affect BTMs and therefore could mask relations between BTM levels and other independent variables. Factors included for univariate regression analyses were study population specific characteristics (e.g. factors related to RRSO and history of breast cancer), bone related characteristics and factors associated with BTMs in literature. Dichotomous variables with N ≥ 5 for each option were included. Multivariate regression analyses were performed with manual conditional stepwise backward inclusion of variables that had a *p*-value < 0.25 in univariate analysis. Comparison of BTM Z-scores between women that were included versus those that were excluded from regression analyses was done by using the Mann-Whitney U test, Chi Square Test or Fisher’s Exact Test when applicable.

Statistical analyses were performed with IBM SPSS Statistics 20 software (SPSS, Chicago, III). *p*-values < 0.05 were considered significant.

## Results

### Study population

**Fig 1:**
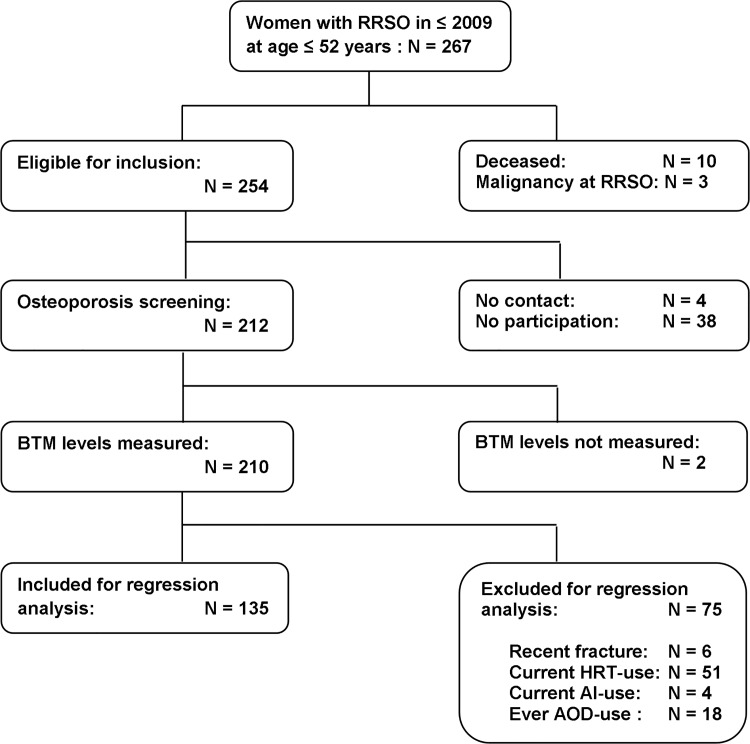
Flowchart of the inclusion process. BTMs were not measured in two women because of logistic reasons.

Of the 254 women eligible for inclusion, 212 women agreed to participate (*Fig 1*). Two women were excluded because blood sampling and thus measurement of BTMs was not possible. The median age at time of study participation was 44 years (IQR 41–49 years; *Table 1*). Median age at RRSO was 42 years (38–46) and median time since RRSO was 5 years (4–8). Of all women, 75 (36%) had a history of fractures, 21 (10%) had fractures at adult age and 16 (8%) had one or more fractures after RRSO. Details on fracture type in women before and after RRSO are provided in S1 Table.

**Table 1:** Study population characteristics (N = 210).

Basic characteristics			Lifestyle characteristics		
Age in years		44 (41–49)	Exercise		169 (81)
Age at RRSO in years		42 (38–46)	Sports		133 (63)
Time since RRSO in years		5 (4–8)	Current smoking		40 (19)
BMI in kg/m^2^		25.9 (22.9–29.5)	Alcohol consumption in units/week		2.5 (0–6)
Parity		2 (1.8–3.0)		> 7 units/week	38 (18)
Menopausal status before RRSO			**Drug use**		
	Premenopausal	176 (84)	Ever use HRT		100 (48)
	Postmenopausal	26 (12)		Current use	51 (24)
	Hysterectomy	8 (4)	Ever use AI		11 (5)
**Oncologic characteristics**				Current use	4 (2)
Mutation status			Ever use tamoxifen		15 (7)
	*BRCA1*	121 (58)		Current use	-
	*BRCA2*	58 (28)	Ever use AOD		18 (9)
	HBOC^a^	31 (15)		Current use	8 (4)
History of breast cancer		78 (37)	Current use of GCS		19 (9)
Chemotherapy		59 (28)	Longterm use of GCS^c^,^d^		8 (4)
Radiotherapy		49 (23)	Current use of calcium		34 (16)
**Bone related characteristics**			Current use of vitamin D3		34 (16)
History of fractures		75 (36)	Current use of multivitamin		26 (12)
	Fracture at adult age^b^	21 (10)	**Laboratory measurements**		
	Fracture after RRSO	16 (8)	Corrected calcium in mmol/L^c^,^e^		2.26 (2.22–2.30)
	Recent fracture	6 (3)	Serum 25OHD in nmol/L		65 (50–82)
BMD LS in grams/cm^2^^c^		0.97 (0.88–1.06)		Low 25OHD^f^	91 (43)
	LS Z-score	0.00 (-0.85–0.85)	Phosphate in mmol/L		1.10 (0.98–1.20)
BMD FN in grams/cm^2^		0.78 (0.71–0.84)	PTH in pmol/L^c^		4.8 (4.0–6.1)
	FN Z-score	0.10 (-0.60–0.80)	TSH in mE/L^c^		1.55 (1.11–2.21)
Osteoporosis (T-score ≤ -2.5)		13 (6)			

Values in median (IQR) or No. (%).

Abbreviations: IQR: interquartile range (i.e. 25th percentile– 75th percentile), RRSO: risk-reducing salpingo-oophorectomy, BMI: body mass index, HBOC: hereditary breast ovarian cancer, BMD: bone mineral density, LS: lumbar spine, FN: femoral neck, HRT: hormonal replacement therapy, AI: aromatase inhibitor, AOD: anti-osteoporotic drugs, GCS: glucocorticosteroids, PTH: parathyroid hormone, TSH: thyroid stimulating hormone.

a. HBOC women had RRSO because of family history of breast or ovarian cancer, 15 had breast cancer, 23 had negative BRCA testing in the family, two were negative and one was not tested for their familial BRCA mutation, four had no BRCA testing and BRCA status of the family was unknown

b. Adult age is ≥ 20

c. Missing values for: BMD LS N = 1, long term use of GCS N = 1, Corrected calcium N = 1, PTH N = 4, TSH N = 26

d. Use of prednisone 7.5 mg or equivalent > 3 months or > 3 oral prednisolone courses per years

e. Calcium was corrected for albumin levels with the following formula: Corrected calcium (mmol/L) = measured total Calcium (mmol/L) + 0.02 (41 –serum albumin [g/L])

f. Low for season: <50 nmol/L October—April; < 75 nmol/L April–October.

For the regression analyses 75 women were excluded because of recent fractures (N = 6) and/or BTM affecting medication (N = 71). Compared to the women included for regression analyses, these excluded women were significantly younger at the time of the study and at time of RRSO, had lower BMIs, had lower absolute BMD in grams/cm^2^ but not BMD Z-score for both LS and FN and had higher calcium and phosphate levels (*S2 Table*).

### Bone formation markers OC and PINP

**Fig 2:**
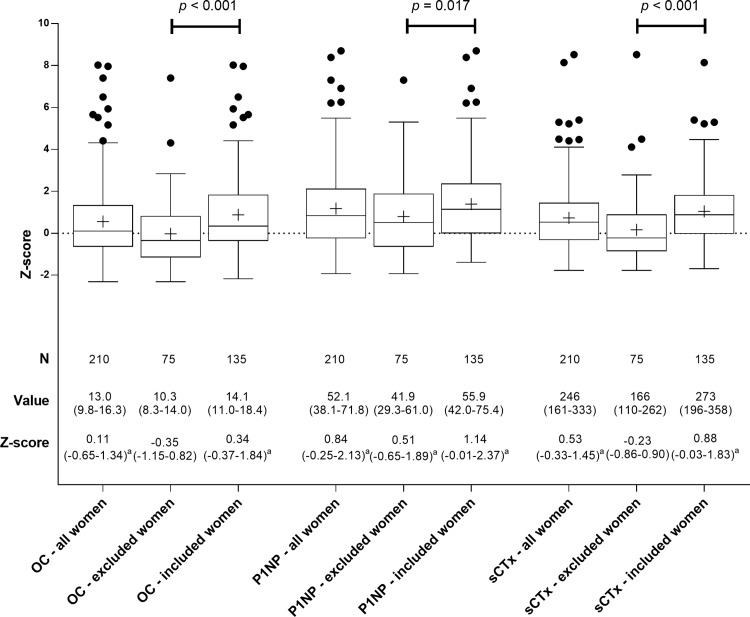
Levels of Osteocalcin, PINP and sCTx after RRSO in all women (N = 210), excluded women with recent fractures or currently using HRT or AI or ever using AOD (N = 75) and women included for regression analyses (N = 135). Box-and whisker plots (Tukey): boxes indicate medians with interquartile ranges; + indicate means; whiskers indicate 1.5 times the interquartile distances; ∙ indicate outliers. a. *p* < 0.05; median Z-scores are compared to a median Z-score of 0.

Median OC level for all women was 13.0 ng/ml (IQR 9.8–15.3). Median OC Z-score was 0.11 (-0.65–1.34; *Fig 2*), which was significantly higher than the theoretical median Z-score of 0 in the reference cohort (*p* = 0.003). Median OC Z-score in women included for regression analysis was significantly higher than in the women excluded for regression analysis (*Fig 2*). Results for univariate analyses are shown in Table 2. In multivariate analysis, OC Z-scores were higher in women who were younger at time of RRSO (β -0.098 per year), had lower femoral neck BMD Z-score (β -0.425 per SD, and had higher corrected serum calcium ((β 5.573 per mmol/L) and phosphate levels (β 3.193 per mmol/L; Table 3).

**Table 2:** Determinants related to BTM Z-scores as assessed by univariate linear regression analyses after exclusion of women with recent fractures, ever AOD use and current HRT or AI use (N = 135).

	Z-score OC			Z-score PINP			Z-score sCTx			Z-score PINP-sCTx		
	β	SE	*p*	β	SE	*p*	β	SE	*p*	β	SE	*p*
**Basic characteristics **												
Age > 50	-0.732	0.317	**0.023**	-1.430	0.306	**0.000**	-0.573	0.247	**0.022**	-0.857	0.280	**0.003**
Age at RRSO (per year)	-0.092	0.032	**0.004**	-0.157	0.031	**0.000**	-0.058	0.025	**0.022**	-0.099	0.028	**0.001**
Time since RRSO (per year)	-0.132	0.051	**0.011**	-0.122	0.052	**0.021**	-0.044	0.041	0.276	-0.078	0.046	0.097
BMI (per kg/m^2^)	-0.057	0.027	**0.038**	-0.019	0.028	0.502	-0.038	0.021	0.080	0.019	0.025	0.454
Parity	-0.047	0.135	0.728	-0.265	0.136	0.054	-0.078	0.105	0.459	-0.187	0.120	0.123
Postmenopausal before RRSO	-0.943	0.454	**0.040**	-0.926	0.461	**0.047**	-0.473	0.357	0.188	-0.453	0.412	0.274
**Oncologic charachteristics **												
History of breast cancer	-0.132	0.331	0.689	-0.161	0.338	0.634	-0.186	0.257	0.471	0.025	0.297	0.933
Chemotherapy	-0.213	0.349	0.541	-0.244	0.356	0.494	-0.295	0.271	0.278	0.051	0.313	0.870
Radiotherapy	-0.204	0.372	0.585	-0.288	0.379	0.449	-0.011	0.290	0.970	-0.277	0.333	0.407
**Bone related characteristics **												
Fracture at adult age^a^	0.079	0.548	0.885	-0.757	0.555	0.175	0.111	0.427	0.795	-0.868	0.485	0.076
Fracture after RRSO	0.669	0.727	0.359	1.116	0.738	0.133	0.386	0.567	0.497	0.730	0.650	0.263
LS Z-score	-0.456	0.141	**0.002**	-0.377	0.144	**0.010**	-0.362	0.109	**0.001**	-0.015	0.132	0.909
FN Z-score	-0.531	0.171	**0.002**	-0.315	0.179	0.080	-0.280	0.136	**0.041**	-0.035	0.159	0.825
Osteoporosis (T-score ≤ -2.5)	-0.263	0.648	0.685	-0.006	0.661	0.993	0.216	0.505	0.670	-0.222	0.580	0.703
**Lifestyle characteristics**												
Exercise^b^	-0.044	0.423	0.917	-0.345	0.430	0.424	0.335	0.328	0.310	-0.680	0.374	0.071
Sports	0.123	0.328	0.708	-0.239	0.334	0.475	0.224	0.255	0.380	-0.464	0.291	0.114
Current smoking	-0.012	0.423	0.978	0.228	0.431	0.597	-0.451	0.327	0.171	0.679	0.374	0.072
Alcohol consumption in units/week	-0.023	0.029	0.428	-0.019	0.030	0.527	0.004	0.023	0.859	-0.023	0.026	0.381
> 7 units/week	-0.218	0.422	0.607	-0.091	0.431	0.833	0.044	0.329	0.893	-0.135	0.379	0.721
**Drug use**												
Past use of HRT	0.598	0.337	0.078	1.010	0.337	**0.003**	0.493	0.262	0.062	0.516	0.302	0.090
Ever use of tamoxifen	-0.270	0.684	0.694	-0.428	0.698	0.541	-0.733	0.530	0.169	0.305	0.613	0.619
Current use of GCS	-0.114	0.530	0.829	-0.305	0.540	0.573	0.339	0.412	0.412	-0.645	0.472	0.174
Current use of calcium	0.440	0.463	0.344	0.138	0.474	0.771	-0.075	0.362	0.836	0.213	0.416	0.609
Current use of vitamin D3	0.504	0.473	0.289	0.163	0.485	0.738	0.066	0.370	0.860	0.097	0.426	0.820
Current use of multivitamin	-0.057	0.455	0.900	-0.769	0.460	0.097	-0.387	0.353	0.274	-0.381	0.406	0.350
**Laboratory measurements**												
Corrected calcium^c^	5.436	2.237	**0.016**	3.241	2.314	0.164	1.173	1.781	0.511	2.068	2.037	0.312
Serum 25OHD	0.011	0.007	0.091	0.005	0.007	0.453	0.007	0.005	0.175	-0.002	0.006	0.748
Low 25OHD^d^	-0.553	0.320	0.087	-0.250	0.330	0.450	-0.412	0.250	0.101	0.162	0.290	0.577
Phosphate	3.166	1.084	**0.004**	1.890	1.130	0.097	1.017	0.867	0.243	0.873	0.999	0.384
PTH	0.076	0.076	0.319	-0.010	0.079	0.897	0.064	0.060	0.287	-0.074	0.069	0.282
TSH	-0.011	0.139	0.940	-0.052	0.142	0.715	0.007	0.105	0.945	-0.059	0.126	0.638

*p*-values were calculated using univariate linear regression analysis. Bold numbers indicate *p* < 0.05. Abbreviations: BTM: bone turnover marker; OC: osteocalcin, PINP: procollagen type I N-terminal propeptide; sCTx: serum C-telopeptide of type I collagen; SE: standard error; RRSO: risk-reducing salpingo-oophorectomy, BMI: body mass index; LS is lumbar spine; FN is femoral neck; HRT: hormonal replacement therapy; AI: aromatase inhibitor; GCS is glucocorticosteroids; PTH is parathyroid hormone; TSH is thyroid stimulating hormone.

a. Adult age is ≥ 20

b. ≥ 30 minutes of exercise a day

c. Calcium was corrected for albumin levels with the following formula: Corrected calcium (mmol/L) = measured total calcium (mmol/L) + 0.02 (41 –serum albumin [g/L])

d. Low for season: < 50 nmol/L October—April; < 75 nmol/L April–October.

**Table 3:** Determinants related to BTM Z-scores as assessed by multivariate linear regression analyses.

	Multivariate analysis on Z-score OC			Multivariate analysis on Z-score PINP			Multivariate analysis on Z-score sCTx			Z-score PINP—sCTx		
	(N = 134)			(N = 135)			(N = 134)			(N = 135)		
	β	SE	*p*	β	SE	*p*	β	SE	*p*	β	SE	*p*
Age at RRSO (per year)	-0.098	0.029	**0.001**	-0.157	0.030	0.000				-0.099	0.028	0.001
Time since RRSO (per year)				-0.121	0.048	0.012						
LS Z-score							-0.373	0.107	0.001			
FN Z-score	-0.531	0.158	**0.001**									
Past use of HRT							0.565	0.250	0.025			
Corrected calcium^a^	5.573	2.024	**0.007**									
Phosphate	3.193	1.002	**0.002**									

*p*-values were calculated using multivariate linear regression analysis with manual conditional stepwise backward inclusion of variables that had a *p*-value < 0.25 in univariate analysis. Bold numbers indicate *p* < 0.05. Abbreviations: BTM: bone turnover marker; OC: osteocalcin, PINP: procollagen type I N-terminal propeptide; sCTx: serum C-telopeptides of type I collagen; SE: standard error; RRSO: risk-reducing salpingo-oophorectomy, LS is lumbar spine; FN is femoral neck; HRT: hormonal replacement therapy.

a Calcium was corrected for albumin levels: Corrected calcium (mmol/L) = measured total calcium (mmol/L) + 0.02 (41 –serum albumin [g/L]).

Median PINP level for all women was 52.1 ng/ml (IQR 38.1–71.8).Median Z-score was 0.84 (-0.35–2.13; *Fig 2*), which was significantly higher than the theoretical median Z-score of 0 in the reference cohort (*p* < 0.001). Median PINP Z-score in women included for regression analysis was significantly higher than in the women excluded for regression analysis (*Fig 2)*. In multivariate analysis, PINP Z-scores were higher in women who were younger at the time of RRSO (β -0.157 per year) and had a shorter time since RRSO (β -0.121 per year; *Table 3*).

### Bone resorption marker sCTx

Median sCTx level for all women was 246 pg/ml (IQR 160–133).Median Z-score of 0.53 (-0.33–1.45; *Fig 2*), which was significantly higher than the hypothetical median Z-score of 0 in the reference cohort (*p* < 0.001). Median sCTx Z-score in women included for regression analysis was significantly higher than in the women excluded for regression analysis (*Fig 2).* In multivariate analysis, sCTx Z-scores were higher in women who had lower lumbar spine Z-scores (β -0.373 per SD) and used HRT in the past (β 0.565 for users compared to non-users; *Table 3*).

### Difference between Z-scores for PINP and sCTx

**Fig 3:**
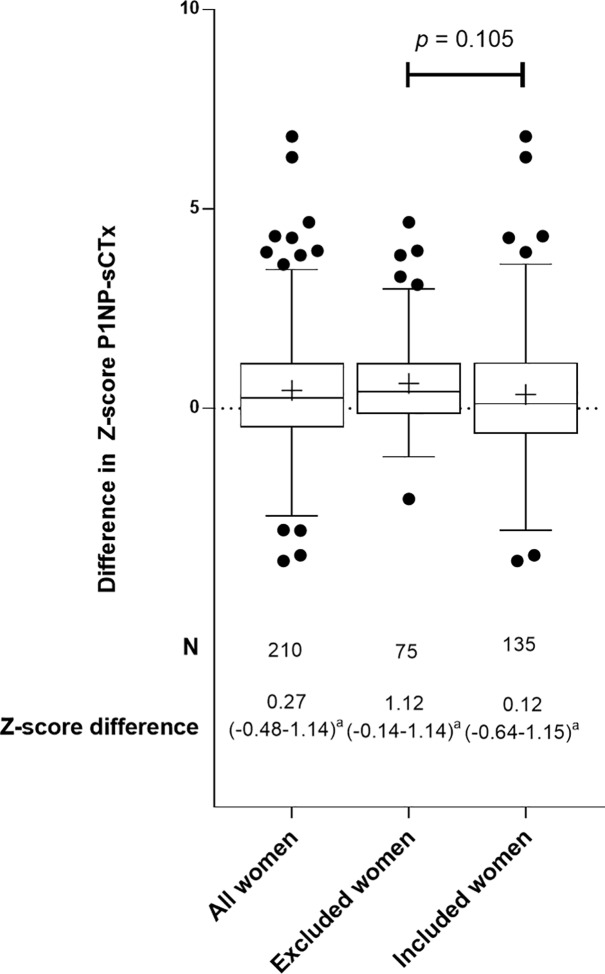
Difference in Z-scores for PINP and sCTx after RRSO in all women (N = 210), excluded women with recent fractures or currently using HRT or AI or ever using AOD (N = 75) and women included for regression analyses (N = 135). Box-and whisker plots (Tukey): boxes indicate medians with interquartile ranges; + indicate means; whiskers indicate 1.5 times the interquartile distances; ∙ indicate outliers. a. *p* < 0.05; difference in Z-scores are compared to a median difference of 0.

The median difference between Z-scores for PINP and sCTx for all women was 0.27 (-0.48–1.14) (*Fig 3*). This difference was similar in the women that were in- and excluded for regression analyses. Younger age at RRSO was correlated with an increasing positive difference between PINP and sCTx Z-score.

## Discussion

Within this consecutive series of 210 women that underwent RRSO before age 53, with a median time after RRSO of 5 years, OC, PINP and sCTx levels were significantly higher than age-matched reference values. After excluding women with recent fractures and those using BTM affecting medication, Z-scores for bone formation markers were higher in women with younger age at time of RRSO. No significant correlation was found between BTM Z-scores and history of fractures or breast cancer.

Our finding that BTMs after RRSO were higher than age-matched reference values is partially in line with the results of Morgante et al. [20], who reported higher plasma OC and urinary deoxypyridoline levels in women with surgical menopause compared to age-matched premenopausal women, but not to age-matched women with natural menopause. In univariate regression analyses, Z-scores for all BTMs were significantly higher in women aged ≤ 50 years compared to women aged > 50, which is in line with the finding that BTM levels after surgical menopause differ more from premenopausal then from natural menopausal women. Also partially in line with our results, are the observations of Ohta *et al*. [19] who reported higher serum levels of type I carboxy-terminal pyridinoline cross-linked telopeptide within three years after surgical menopause compared to natural menopause, but not after three years and not for other bone resorption markers. In this study, women more than three years after surgical menopause appeared to be older and had a longer time interval after menopause than our study population. Garcia-Perez et al. [21] showed similar levels of serum OC and urinary N-telopeptide of type I collagen in women with surgical and natural menopause, but in that study women with natural menopause were significantly older than women with surgical menopause, thus it can not be ruled out that women with surgical menopause had a higher bone turnover than women after natural menopause matched for age.

Several factors were significantly correlated with BTMs in multivariate analyses. Most importantly, there was an inverse correlation between age at RRSO and BTM Z-scores for OC and PINP. This might indicate that every years a woman is younger at time of RRSO, is associated with significantly higher bone formation marker levels after correction for chronological age. To our knowledge, this association was not investigated in previous studies. The finding might indicate a more profound effect of RRSO on bone turnover when surgery is performed at a younger age. Also, there was a positive correlation between OC Z-scores and corrected serum calcium levels, which is in contrast with earlier findings [26,27] and a positive correlation between OC levels and phosphate levels, which is in line with some [26], but not all earlier findings [27]. PINP Z-scores were negatively correlated with time since RRSO, which might indicate that with a longer time interval after RRSO BTMs will normalize to age-appropriate values. Higher OC and sCTx Z-scores were correlated with lower BMD, which indicates that high bone turnover is associated with bone loss after RRSO. This was seen before for OC [20] and sCTx [14].

Last, we found a correlation between past HRT use and higher sCTx Z-score, which is in line with earlier findings that after cessation of HRT use, sCTx levels increase significantly [28]. In contrast, current HRT use is associated with a significant increase in BMD and decrease in BTMs [29,30]. In our study population, the 24% of the included women using HRT had significantly lower BTM Z-scores and were excluded for regression analysis. Although, HRT is effective in improving BMD and preventing fractures, prescribing HRT after the age of natural menopause is not advised in BRCA mutation carriers with RRSO after the age of natural menopause [31].

Treatment with AI increases bone turnover and decreases BMD [32], and women using AI often receive AOD for prevention, thus we excluded these women from the regression analyses. Of the women included for regression analyses, 53 (39%) had a history of breast cancer, but this was not correlated with BTMs. There were no women with clinical symptoms of bone metastases. However, because of the exclusion of a significant number of breast cancer patients from the regression analyses, we cannot exclude an effect of history of breast cancer on BTMs.

The median difference between Z-scores for PINP and sCTx reflects the difference in formation and resorption of collagen relative to age-matched controls. This difference can be considered as a marker for absolute bone turnover. We found a median difference of 0.12 (-0.64–1.15), which was significantly higher than 0 in the reference population. There was an inverse relation between age at RRSO and the difference between Z-score for PINP and sCTx. The clinical implications of this finding are unknown; however it does not suggest an imbalance favouring bone resorption after RRSO. As there are no earlier studies that have reported on this difference between PINP and sCTx Z-scores, there is a need for more studies on the clinical interpretation of findings on the relative difference between these markers.

Strengths of this study are the unselected sample that is representative of a patient population with surgical menopause due to RRSO, and the large study population compared to earlier studies. Furthermore, BTMs were presented as Z-scores to increase comparability with other studies, as was advised by Vasikaran et al. [12]. Limitations of the study are that we were not able to fully correct for several factors that influence BTMs, such as diurnal variation and the timing of food intake. As it is known that BTMs decrease during the afternoon and after ingestion of a meal [12], not correcting for these factors could have attenuated elevated BTMs. The percentage of women with surgical menopause in the reference cohort was unknown, but the prevalence of surgical menopause in the general population is low. In addition, women with RRSO before age 53 were assumed to be premenopausal before RRSO, but some reported to be postmenopausal at that time. Women who were postmenopausal before RRSO had lower OC and PINP Z-scores in univariate analyses. Both factors might have caused underestimation of the effect of RRSO on BTMs.

The clinical implications of BTM elevation after RRSO are unknown, but elevated BTMs have been shown to predict elevated fracture risk in longitudinal population studies, independent of BMD [12,13]. This might tone down the reassuring findings of our previous study in this study population, which showed that BMD and fracture incidence after RRSO were comparable to population data [11]. Although we did not find a correlation between BTMs and fracture incidence in this cross-sectional study, longitudinal studies are needed to evaluate the long-term clinical implications of elevated BTMs after RRSO.

In conclusion, this study shows that after a median time of 5 years after premenopausal RRSO, BTMs are elevated compared to age-matched reference values, especially in women with RRSO at younger age. In this cross-sectional study, no relation between elevated BTMs and fracture incidence was shown. However, as elevated BTMs predict elevated fracture risk in the general population, prospective studies are required to evaluate the long-term clinical implications of elevated BTMs after RRSO.

## Supporting Information

S1 Dataset SPSS file containing the data underlying the findings described in this manuscript. (SAV)

S1 Table Details on fractures reported in the study population (N = 210). (DOC)

S2 Table Characteristics of the women who were in- and excluded for regression analysis on BTMs after RRSO. Women ever using AOD, currently using AI or HRT or with recent fractures were excluded.
(DOC)
