# Supplementary material for: Elevated Bone Turnover Markers after Risk-Reducing Salpingo-Oophorectomy in Women at Increased Risk for Breast and Ovarian Cancer
Source: PLoS One. 2017 Jan 6;12(1):e0169673. doi: 10.1371/journal.pone.0169673 (PMC5218401; doi:10.1371/journal.pone.0169673)
Supplement: S1 Table — (DOC) [file pone.0169673.s002.doc]

**S1 Table: Details on fractures reported in the study population (N = 210)**

|  | **Before RRSO** | **After RRSO** |
| --- | --- | --- |
| **Women reporting fractures - No. (%)** | 64 (30) | 16 (8) |
| **Fractures – Total (No).** | 105 | 23 |
| **Fracture types - No. (%)** |  |  |
| **Hip** | 0 | 0 |
| **Wrist** | 16 (15) | 2 (9) |
| **Vertebra** | 4 (4) | 3 (13) |
| **Nose** | 6 (6) | 0 |
| **Clavicle** | 2 (2) | 2 (9) |
| **Rib** | 5 (5) | 0 |
| **Sternum** | 1 (1) | 0 |
| **Arm** | 10 (10) | 1 (4) |
| **Elbow** | 6 (6) | 3 (13) |
| **Hand** | 4 (4) | 1 (4) |
| **Pelvis** | 1 (1) | 1 (4) |
| **Leg** | 9 (9) | 0 |
| **Knee** | 1 (1) | 0 |
| **Ankle/foot** | 17 (16) | 4 (17) |
| **Toe/finger** | 23 (22) | 6 (26) |
