# Supplementary material for: Elevated Bone Turnover Markers after Risk-Reducing Salpingo-Oophorectomy in Women at Increased Risk for Breast and Ovarian Cancer
Source: PLoS One. 2017 Jan 6;12(1):e0169673. doi: 10.1371/journal.pone.0169673 (PMC5218401; doi:10.1371/journal.pone.0169673)
Supplement: S2 Table — Women ever using AOD, currently using AI or HRT or with recent fractures were excluded. (DOC) [file pone.0169673.s003.doc]

**S2 Table: Characteristics of the women who were in- and excluded for regression analysis on BTMs after RRSO. Women ever using AOD, currently using AI or HRT or with recent fractures were excluded.**

| **Basic characteristics** | | **Included women (N=135)** | **Excluded women (N=75)** | ***p*-value** | **Lifestyle characteristics** | | | **Included women (N=135)** | **Excluded women (N=75)** | ***p*-value** |
| --- | --- | --- | --- | --- | --- | --- | --- | --- | --- | --- |
| Age in years | | 50 (47-54) | 46 (42-50) | **<0.001** | Exercise | | | 111 (82) | 58 (77) | 0.392 |
| Age at RRSO in years | | 44 (40-47) | 39 (36-42) | **<0.001** | Sports | | | 79 (59) | 54 (72) | 0.052 |
| Time since RRSO in years | | 5 (4-8) | 6 (4-8) | **0.728** | Current smoking | | | 24 (18) | 16 (21) | 0.530 |
| BMI in kg/m^2^ | | 26.2 (23.3-30.0) | 24.8 (22.3-29.1) | **0.030** | Alcohol consumption in units/week | | | 3 (0-6) | 2 (0-6) | 0.926 |
| Parity | | 2 (1-3) | 2 (2-3) | 0.862 |  | | > 7 units/week | 24 (18) | 14 (19) | 0.873 |
| Menopausal status before RRSO | |  |  | 0.137 | **Drug use** | | | | | |
|  | Premenopausal | 109 (81) | 67 (89) |  | Ever use HRT | | | 46 (34) | 54 (72) | **<0.001** |
|  | Postmenopausal | 20 (15) | 6 (8) |  |  | Current use | | N/A | 51 (68) | N/A |
|  | Hysterectomy | 6 (4) | 2 (3) |  | Ever use AI | | | 4 (3) | 7 (9) | 0.058 |

| **Oncologic characteristics** |  | Current use | N/A | 4 (5) |  |
| --- | --- | --- | --- | --- | --- |

| Mutation status | |  |  | 0.153 | Ever use tamoxifen | | 8 (6) | 7 (9) | 0.358 |
| --- | --- | --- | --- | --- | --- | --- | --- | --- | --- |
|  | *BRCA1* | 72 (53) | 49 (65) |  |  | Current use | - | - |  |
|  | *BRCA2* | 39 (29) | 19 (25) |  | Ever use AOD | | N/A | 18 (24) | N/A |
|  | HBOC | 24 (18) | 7 (9) |  |  | Current use | N/A | 8 (11) | N/A |
| History of breast cancer | | 53 (39) | 25 (33) | 0.394 | Current use of GCS | | 14 (10) | 5 (7) | 0.370 |
| Chemotherapy | | 42 (31) | 17 (23) | 0.192 | Longterm use of GCS^b^,^c^ | | 4 (3) | 4 (5) | 0.396 |
| Radiotherapy | | 34 (25) | 15 (20) | 0.395 | Current use of calcium | | 19 (14) | 15 (20) | 0.264 |

| **Bone related characteristics** | Current use of vitamin D3 | 18 (13) | 16 (21) | 0.132 |
| --- | --- | --- | --- | --- |

| History of fractures | | 43 (32) | 32 (43) | 0.117 | Current use of multivitamin | | 20 (15) | 6 (8) | 0.151 |
| --- | --- | --- | --- | --- | --- | --- | --- | --- | --- |
|  | Fracture at adult age^a^ | 13 (10) | 8 (11) | 0.810 | **Laboratory measurements** | | | | |
|  | Fracture after RRSO | 7 (5) | 9 (12) | 0.073 | Corrected calcium in mmol/L^b,d^ | | 2.27 (2.23-2.32) | 2.23 (2.19-2.29) | **0.003** |
|  | Recent fracture | N/A | 6 (8) | N/A | Serum 25OHD in nmol/L | | 64 (49-83) | 66 (54-79) | 0.431 |
| BMD LS in gr/cm^2b^ | | 0.95 (0.86-1.04) | 0.99 (0.90-1.08) | **0.029** |  | Low 25OHD^e^ | 64 (47) | 27 (36) | 0.110 |
|  | LS Z-score^e^ | -0.10 (-0.90-0.83) | 0.10 (-0.70-0.90) | 0.371 | Phosphate in mmol/L | | 1.13 (1.04-1.21) | 1.00 (0.88-1.12) | **<0.001** |
| BMD FN in gr/cm^2^ | | 0.76 (0.69-0.83) | 0.80 (0.73-0.88) | **0.017** | PTH in pmol/L^b^ | | 5.2 (4.1-6.4) | 4.55 (3.87-5.60) | 0.050 |
|  | FN Z-score | 0.00 (-0.60-0.70) | 0.20 (-0.40-0.80) | 0.329 | TSH in mE/L^b^ | | 1.54 (1.09-2.21) | 1.59 (1.19-2.21) | 0.371 |
| Osteoporosis (T-score ≤ -2.5) | | 9 (7) | 4 (5.3) | 0.775 |  | |  |  |  |

Values in median (IQR) or No. (%). Comparisons by Mann-Whitney U test for non-parametric data and Chi Square or Fisher’s Exact Test for dichotomous or categorical data.

Abbreviations: IQR: interquartile range (i.e. 25th percentile – 75th percentile), RRSO: risk-reducing salpingo-oophorectomy, BMI: body mass index, HBOC: hereditary breast ovarian cancer, BMD: bone mineral density, LS: lumbar spine, FN: femoral neck, HRT: hormonal replacement therapy, AI: aromatase inhibitor, AOD: anti-osteoporotic drugs, GCS: glucocorticosteroids, PTH: parathyroid hormone, TSH: thyroid stimulating hormone.

a. Adult age is ≥ 20; b. Missing values for: BMD LS N=1, longterm use of GCS N=1, Corrected calcium N=1, PTH N=4, TSH N=26; c. Use of prednisone 7.5 mg or equivalent > 3 months or > 3 oral prednisolone courses per years; d. Calcium was corrected for albumin levels with the following formula: Corrected calcium (mmol/L) = measured total calcium (mmol/L) + 0.02 (41 – serum albumin [g/L]); e. Low for season: <50 nmol/L October - April; <75 nmol/L April – October.
